# Phytochemical Properties and Nutrigenomic Implications of Yacon as a Potential Source of Prebiotic: Current Evidence and Future Directions

**DOI:** 10.3390/foods7040059

**Published:** 2018-04-12

**Authors:** Yang Cao, Zheng Feei Ma, Hongxia Zhang, Yifan Jin, Yihe Zhang, Frank Hayford

**Affiliations:** 1 Department of Health Promotion, Pudong Maternal and Child Health Care Institution, Shanghai 201399, China; 2 Department of Public Health, Xi’an Jiaotong-Liverpool University, Suzhou 215123, China; Yifan.Jin14@student.xjtlu.edu.cn; 3 School of Medical Sciences, Universiti Sains Malaysia, Kota Bharu 15200, Kelantan, Malaysia; 4 Department of Food Science, University of Otago, Dunedin 9016, New Zealand; zhanghongxia326@hotmail.com; 5 Division of Medicine, School of Life and Medical Sciences, University College London, London WC1E6BT, UK; yihe.zhang.16@ucl.ac.uk; 6 Department of Nutrition and Dietetics, School of Biomedical and Allied Health Sciences, College of Health Sciences, University of Ghana, Accra P.O. Box LG 25, Ghana; feahayford220580@gmail.com

**Keywords:** yacon, *Smallanthus sonchifolius*, prebiotic, fructooligosaccharides, underutilized

## Abstract

The human gut is densely populated with diverse microbial communities that are essential to health. Prebiotics and fiber have been shown to possess the ability to modulate the gut microbiota. One of the plants being considered as a potential source of prebiotic is yacon. Yacon is an underutilized plant consumed as a traditional root-based fruit in South America. Yacon mainly contains fructooligosaccharides (FOS) and inulin. Therefore, it has bifidogenic benefits for gut health, because FOS are not easily broken down by digestive enzymes. Bioactive chemical compounds and extracts isolated from yacon have been studied for their various nutrigenomic properties, including as a prebiotic for intestinal health and their antimicrobial and antioxidant effects. This article reviewed scientific studies regarding the bioactive chemical compounds and nutrigenomic properties of extracts and isolated compounds from yacon. These findings may help in further research to investigate yacon-based nutritional products. Yacon can be considered a potential prebiotic source and a novel functional food. However, more detailed epidemiological, animal, and human clinical studies, particularly mechanism-based and phytopharmacological studies, are lacking for the development of evidence-based functional food products.

## 1. Introduction

A focus on the role of gut microbiota to improve health and prevent disease has attracted intense interest in identifying dietary strategies to modulate the gut microbiota. One such dietary strategy includes the intake of prebiotics and dietary fiber, because they can be metabolized by the gut microbiota. One potential candidate for prebiotics is yacon, which has an abundance of free sugar and fructans with low polymerization (i.e., fructooligosaccharides (FOS)).

Yacon (*Smallanthus sonchifolius*), an underutilized crop, belongs to the family Asteraceae [1]. Originating from the Andean region of South America, yacon is a little known perennial herb that generally takes 6 to 12 months to reach maturity. The aerial stems of yacon can reach about 2.5 m in height [1]. The roots of yacon have a similar appearance to sweet potato, and their weight is about 500 g each. Each yacon plant has about 20 units and can yield >10 kg of roots [2]. The roots of yacon are about 10 cm thick and 20 cm long, in various sizes and shapes. Yacon tuber roots are usually eaten as fruits [3]. The edible roots of yacon are juicy and sweet like an apple and can be consumed either raw or cooked. Its leaves can also be used to brew a medicinal tea [4].

Since the pre-Incan period, yacon has been cultivated and consumed. Its low nutritive value might be one of the reasons why it is being neglected, especially by older Andean agronomists [1]. The scientific community paid little attention to yacon until the 1980s, except in Peru and Japan [5]. Yacon products can range from pickles to dried flakes. Although yacon has a sweet taste and is juicy, it is considered a food with low energy value because of the low-molecular-weight carbohydrate FOS [1]. The roots of yacon mainly contain fructans, and its leaves have been reported to possess putative medicinal compounds. Yacon can provide fiber and low calories for consumers who have an inactive lifestyle with excess intake of fats and carbohydrates. Also, the roots of yacon lack starch, which might be beneficial for the diets of diabetes patients [6]. Therefore, yacon actually has great potential to be a useful plant species.

Its growth and cultivation have spread widely to several countries, such as the Czech Republic, China, Brazil, Japan, Italy, and New Zealand, in recent years due to its presumed physiological benefits and high adaptation to different cultivation environments [3]. The global expansion of yacon cultivation and marketing was further motivated after studies reported on the health benefits of consuming yacon, such as the antioxidant activity associated with its phenolic compounds and the reduction of blood glucose level attributed to its carbohydrate profile [3,6].

Several bioactive compounds were found in both the roots and leaves of yacon, including polyphenol compounds, fructans, and phytoalexins, which show antioxidant, prebiotic, and antimicrobial properties [7,8]. Carbohydrates are stored in yacon in the form of β-(2→1) FOS that can help to prevent constipation and reduce the concentrations of blood glucose and lipids [9]. These functional properties could help people maintain health and reduce the risk of chronic diseases [10,11]. This review will analyze the accumulated evidence on the phytochemical compounds and nutrigenomic properties of yacon, paying special attention to its role as a prebiotic.

### Search Strategy

An electronic literature search was conducted using Cochrane Library, Medline (OvidSP), Google Scholar, and PubMed through January 2018. Additional articles were identified from references located in the retrieved articles. Our search strategy included combinations of the following using Boolean markers: *Smallanthus sonchifolius*, yacon, health, prebiotic, phytochemicals, and fructans. The search was restricted to experimental, epidemiological, and clinical studies published in English that address the phytochemical constituents and nutrigenomic properties of yacon. Our work will also add to the current understanding of some of the bioactive compounds and nutrigenomic properties of yacon that were covered adequately in the previous reviews [2].

## 2. Phytochemical Compounds

### 2.1. Roots/Tubers

Table 1 shows the chemical composition of fresh yacon root. There are three main substances in fresh yacon root: water (>70%), carbohydrates (the major proportion of the dry matter), and protein [12]. Carbohydrates in yacon root contain glucose, fructose, sucrose, and FOS. Among them, FOS are considered the predominant saccharides [4]. FOS are natural food components found in many plants. However, FOS concentrations in the roots of yacon are highest compared to other plants [13]. The chemical structure of FOS has 2 to 10 fructose molecules connected with a β-(1,2) glucosidic bond and 1 glucose molecule linked with α-(1,2) bond [14]. FOS are stable under conditions of pH > 3 and temperature up to 140 °C [15]. The major FOS in yacon include nystose, 1-kestose, and 1-fructofuranosyl nystose [16]. The carbohydrate content in yacon can be influenced by the location of cultivation, season of growing, and time and temperature of postharvest storage. With increased time after harvesting, fructans in yacon rapidly depolymerize to mono- and disaccharides by fructan hydrolase. Under a low temperature of postharvest storage (~10 °C), this conversion speed is slower [1]. FOS is nondigestible in the upper gastrointestinal tract before going through fermentation in the large intestine. The small intestine does not have enzymes to hydrolyze the glucosidic bonds in FOS. Studies have shown that FOS can be fermented by most *Bifidobacterium* strains and some *Lactobacillus* strains, healthy beneficial bacteria that naturally exist in the colon [17,18]. A study by Pedreschi et al. [17] indicated that both *Lactobacillus* and *Bifidobacterium* strains utilized GF2 in root extracts of yacon, while *Bifidobacterium* utilized molecules with longer FOS chains. Consumption of FOS could produce short-chain fatty acids and lead to an increase in *Bifidobacteria* [19,20,21]. A clinical study by Guigoz [22] showed that FOS consumption could modulate intestinal microbiota and have a beneficial effect in improving health outcomes. Taken together, FOS are considered as a prebiotic that meets the criteria defined by the Food and Agriculture Organization (FAO) on prebiotics [23]. Furthermore, FOS have been reported to increase bone density and absorption of magnesium and calcium [24,25,26]. In addition, free fructose is naturally present in vegetables and fruits, including yacon, so its intake is an unavoidable consequence of eating a healthy diet. Only when fructose intake is excessive does it have deleterious metabolic effects in humans [27].

In a study by Goto et al. [12], researchers purified and confirmed the presence of oligosaccharides in the roots of yacon as β-(2→1) with terminal sucrose, which are inulin-type oligofructans, using enzymatic, ^13^C-nuclear magnetic resonance (NMR), and methylation methods. Yan et al. [6] showed the presence of chlorogenic acid and tryptophan in the roots of yacon using NMR and mass spectrometry. Another study, by Takenaka et al. [28], identified five caffeic acid derivatives in the roots of yacon using spectroscopic methods. These compounds were 2,5-dicaffeoylaltraric acid, 3,5-dicaffeoylquinic acid, chlorogenic acid (3-caffeoylquinic acid), 2,4- or 3,5-dicaffeoylaltraric acid, and 2,3,5- or 2,4,5-tricaffeoylaltraric acid [28].

In addition, flavonoids were found only in acid-hydrolyzed yacon tubers and were found to have a relationship with lipid peroxidation, acetylcholinesterase, and butyrylcholinesterase inhibition [5,29]. A study by Simonovska et al. [5] also showed the presence of the flavonoid quercetin, caffeic acid, and ferulic acid using thin-layer chromatography in the acid hydrolysis of yacon tubers. Also, yacon root contains small amounts of vitamins and minerals. Among them, vitamin C and potassium are the most abundant nutrients [15]. Tryptophan, known as a precursor of serotonin and melatonin, is the most abundant amino acid in yacon root [6]. Tryptophan has antioxidant properties. It has been observed that tryptophan is more likely to eliminate free radicals from the oxidative damage of low-density lipoprotein compared with melatonin [30]. However, tryptophan has less antioxidant activity than chlorogenic acid by 1,1-diphenyl-2-picrylhydrazyl (DPPH) assay [6].

### 2.2. Leaves and Flowers

Lin et al. [7] extracted and reported antibacterial compounds including 8β-tigloyloxymelampolid-14-oic acid methyl ester, melampolide-type sesquiterpene lactones, and 8β-methacryloyloxymelampolid-14-oic acid methyl ester, uvedalin, sonchifolin, fluctuanin, melampolides, and enhydrin from yacon leaves. Simonovska et al. [5] also reported the presence of ferulic acid in yacon leaves using thin-layer chromatography.

Yacon is also rich in polyphenols. A study by Hondo et al. [31] showed that yacon juice had 850 ppm of phenolic compounds. A higher polyphenol concentration is usually found in leaves and stems. Chlorogenic, caffeic, ferulic, and protocatechuic acids in tuber and leaf extracts of yacon have also been detected by thin-layer chromatographic screening [5]. Among them, chlorogenic and caffeic acid derivatives are the main polyphenols in yacon, the former at a higher concentration [6]. One investigation of phenolic compounds of yacon found that it contained five caffeic acid derivatives [28]. Once yacon tissue is exposed to the air, it will darken rapidly. The browning reaction is due to a condensation reaction of phenolic compounds with the enzymatic polymerization of polyphenols and amino acids [4]. Polyphenols in yacon leaves provide an acrid and astringent flavor and characteristic odor. Polyphenols are highly related to superoxide radical, DPPH radical, and nitric oxide scavenging activities, which indicates that these compounds have antioxidant properties and may play an important role in lowering the risk of cancer, cardiovascular disease (CVD), atherosclerosis, and diabetes [10,29,32,33].

Sonchifolin, polymatin B, uvedalin, two melampolide-type sesquiterpene lactones, and enhydrin were isolated from yacon leaf extract as an antifungal substance; among them, sonchifolin showed high antimicrobial activity against *Pyricularia oryzae* [7,34]. Moreover, it was reported that there is a high proportion of *ent*-kaurenic acid and kaurene derivatives in yacon leaves and they are involved in the protective mechanism of the glandular trichome exudates [35]. Table 2 shows an overview of major phytochemical compounds in yacon.

## 3. Nutrigenomic Properties of Yacon

Since yacon is an underutilized plant, limited studies have been conducted to determine its nutrigenomic properties [1]. Therefore, there is no conclusive information on the relationship between yacon consumption and its nutrigenomic value. Although the literature shows an association with the nutrigenomic properties [1,39], a causal relationship between yacon and observed health outcomes has not been firmly established. Similar to nutrigenomic properties reported in other plants [40,41,42], the findings of such studies on yacon should be interpreted with caution. Table 3 shows the major nutrigenomic properties of yacon. Yacon has been investigated for its various nutrigenomic properties using epidemiological, animal, and human clinical studies. In addition, the possible mechanisms underlying some of them have been determined and are discussed in the following section.

### 3.1. Beneficial Effects on Intestinal Health

Colorectal cancer (CRC) is the third leading cause of cancer deaths globally [52]. Many risk factors are linked with the occurrence of this disease. Sporadic lifestyle and dietary habits are the main risk factors for most CRC cases [53,54]. Constipation is positively related with an increased risk of colon cancer [55]. In one study, intestinal transit time was significantly decreased, with a slight increase of stool frequency and a tendency for softer stools, after the consumption of 20 g yacon syrup for 2 weeks among healthy participants (*n* = 16) [8]. An improvement in bowel movements was found in a study of constipated elderly patients (*n* = 5) [56] and a study of women with a history of constipation (*n* = 55) [37] that used the same dose of 0.14 g FOS/kg of body weight. These findings [37,56] suggest that foods rich in FOS such as yacon may improve constipation.

A study on 1,2-dimethylhydrazine (DMH)-induced models of colon carcinogenesis in rats showed that yacon and a symbiotic formulation (yacon plus *Lactobacillus acidophilus*) were associated with a reduction of cell proliferation and tumor multiplicity [57]. Similar findings were found in a more recent study, which also showed that aqueous extracts of yacon significantly decreased DNA damage in leukocytes of DMH-induced rats [15].

The mechanisms of improving intestine health might be due to high amounts of FOS in yacon. FOS can stimulate the growth of *Bifidobacteria* to inhibit the growth of pathogenic bacteria. In addition, increasing short-chain fatty acids (SCFAs) produced by FOS fermentation can activate the immune response, lower the pH in the colon, and promote the excretion of amine and ammonia [58]. With reference to carcinogenesis, SCFAs reduce cellular proliferation and cause apoptosis. Studies in rats showed that butyrate slowed down the progress of preneoplastic aberrant crypt foci lesions and postponed the development of tumors [57,59,60].

In an intervention study investigating the effect of yacon flour on nutritional status and immune response biomarkers, Vaz-Tostes et al. [61] reported that preschool children aged 2 to 5 years had an improved intestinal immune response, as shown by an increase in the concentration of serum interleukin (IL)-4 and secretory IgA (sIGA) after the intervention. However, no improvement was seen in the biomarkers of zinc and iron in children [61].

### 3.2. Hypoglycemia Effect

Several studies have shown that yacon has a beneficial effect on reducing blood sugar [43,44,45]. For example, in an animal study, after 30 days, the glucose levels of streptozotocin (STZ)-induced diabetic rats significantly decreased when the rats were treated with 2% yacon tea administered ad libitum [43]. Meanwhile, the plasma insulin level was improved in the treated group as well [43]. These findings were supported by another research study that observed a significant reduction of glycemia in STZ-induced nondiabetic and diabetic rats when they were fed leaf extracts of yacon obtained by hydro-ethanolic extraction [44]. However, when using extracts of yacon obtained by other extract solutions, no hypoglycemic effects were reported, implying that the method of obtaining the extracts was noteworthy. Similar results were found when the experimental material was replaced by dried root extracts [45].

In a 120-day, double-blind, placebo-controlled human intervention study, the consumption of yacon syrup significantly decreased the homeostasis model assessment for insulin resistance and fasting serum insulin in women who were dyslipidemic, premenopausal, and obese [37]. Among elderly individuals, a 9-week intake of freeze-dried powder of yacon was associated with lower serum glucose levels [11]. No significant changes were reported for insulin-stimulated glucose metabolism and fasting plasma glucose of healthy participants when consuming 20 g FOS per day [62].

### 3.3. Hypolipidemic Effect

A study by Genta et al. [47] reported that oral intake of dried yacon root flour for 4 months significantly reduced serum triacylglycerol (TG) levels in normal rats. Their findings were corroborated by a study that observed significant decreases in serum TG levels and very-low-density lipoprotein (VLDL) in STZ-induced diabetic rats treated with yacon flour for 90 days [48]. Interestingly, the low dose of FOS (340 mg/kg) showed more hypolipidemic effect than the high dose (6800 mg/kg). Moreover, Oliveira et al. [46] found that the concentrations of high-density lipoprotein (HDL) cholesterol and total cholesterol were significantly improved in male Wistar rats fed yacon extracts for 14 days. Another study [63] reported that yacon-supplemented diabetic rats had lower malondialdehyde levels in both liver and kidney. Also, yacon-supplemented diabetic rats had lower hepatic dismutase and catalase activity than the controls [63].

Overall, the results in animal studies [46,47,48] seem to be more convincing, while human studies are more controversial [37]. As mentioned previously, a significant reduction of low-density lipoprotein (LDL) was seen in the study of mildly dyslipidemic premenopausal women [37]. These findings were in accordance with results indicating that the hypolipidemic effect of inulin-type fructans is mostly observed in dyslipidemia patients [64]. However, in a double-blind, placebo-controlled study, no reduction in serum lipid levels was reported in an elderly population (*n* = 72) supplemented with freeze-dried yacon powder for 9 weeks [11].

### 3.4. Anti-Inflammatory Effect

Studies have demonstrated that yacon also possesses anti-inflammatory action [7,34,49,50,65,66]. For example, yacon leaf extract might be used as a promising therapeutic agent, especially in topical applications. It is suggested that the anti-inflammatory activity is associated with sesquiterpene lactones (STLs) [7,49], which are found in higher concentrations in the leaves of yacon [34,49,67]. Uvedalin, enhydrin, sonchifolin, and polimatin B are the main STLs detected in yacon leaves [50]. Enhydrin and uvedalin, for instance, have anti-inflammatory properties, as shown by their inhibition of transcription factor NF-κB [50]. A study by Oliveira et al. [49] reported that yacon extract had topical anti-inflammatory and anti-edematous activity, because it reduced edema and neutrophil migration to inflammatory sites when administered to adult male BALB/c mice used as test subjects. This activity may be an important part of the anti-inflammatory action of the extract, exerting some effects on inflammatory mediators, thus demonstrating that yacon leaf extract possesses topical anti-edematous activity in vivo and can be developed as a topical anti-inflammatory agent [49].

### 3.5. Antioxidant Activity

Oxidative stress is suspected to be involved in many chronic diseases, including neurodegenerative diseases, cardiovascular diseases, and certain age-related cancers [36]. Several studies have reported on the presence of phenolic compounds, including caffeic acid, chlorogenic acid, and ferulic acid, which are known to be natural dietary antioxidants, in leaf and tuber extracts of yacon [5,9,28,33]. A study by Oliveira et al. [68] reported that male Wistar rats fed yacon extract had a significant reduction in the serum levels of cardiac markers and an increase in antioxidant defense. In a study investigating the antioxidant properties of sterilized yacon tuber flour, the antioxidant activity of yacon extract was tested by biological assays to determine the effects of protection on directly exposed and phagic DNA [36]. The study results showed that yacon extract had antioxidant activity in protecting DNA from oxidative degradation in both situations, which was contributed by its phenolic compound composition [36].

In a study determining the in vivo antioxidant action of yacon extracts [69], when rat hepatocyte primary cultures were preincubated with yacon leaf extracts before oxidative damage induced by allyl alcohol and tert-butyl hydroperoxide, the toxic effect was less pronounced and the hepatocytes retained high viability. This study indicated that all yacon extracts tested had significant effects on radical scavenging and strong protective effects against oxidative damage to rat hepatocytes [69]. In addition, the study suggested that yacon extracts had an effect on reducing hepatic glucose production, which might contribute to the prevention and treatment of diabetes [69].

### 3.6. Antimicrobial Properties

The cultivation of yacon usually requires almost no pesticides, suggesting that it naturally possesses antimicrobial substances [7]. For example, Inoue et al. [34] first isolated a new antifungal melampolide and three known melampolides from yacon extracts as fungicidal compounds against *P. oryzae*.

Lin et al. [7] also found six melampolide-type sesquiterpene lactones, which were categorized as antibacterial compounds based on their inhibition of *B. subtilis*. A study by Padla et al. [51] investigated one of the antimicrobial compounds isolated from yacon extracts, ent-kaurenoic, which was shown to be active against gram-positive organism (*S. aureus*, *S. epidermidis*, and *B. subtilis)* at the lowest concentration (1000 μg/mL). The authors [51] also suggested that yacon extract has potential protective effects on bacterial skin infections due to its anti-staphylococcal properties. However, more evidence is needed to establish the antimicrobial effects of yacon, because little is known about the antimicrobial effects of yacon on the gut microbiota.

### 3.7. Beneficial Effects on Minerals Balance

Since yacon contains high concentration of fructans, especially FOS, which are regarded as prebiotic ingredients, FOS could be selectively fermented by the gut microbiota in the large intestine. Consequently, this will result in increased levels of short chain fatty acids (SCFAs). SCFAs have an effect on lowering the luminal pH, thereby increasing the solubility of minerals and absorption in the large intestine [70,71].

Lobo et al. [72] conducted a study in growing rats to evaluate the effects of yacon flour consumption on calcium and magnesium balance and bone health. Their results showed that taking yacon flour as a dietary supplement significantly improved intestinal absorption and calcium and magnesium balance, resulting in higher bone mineral retention and stronger bone structural properties than the control group. Nevertheless, increasing the concentration of FOS to 5% or 7.5% in yacon flour fed to rats was related to a significant increase in calcium absorption and calcium and magnesium balance. Although all bone parameters showed an increase in the yacon-supplemented group compared with the control group, only peak loads and stiffness were observed to significantly differ between the groups [72], which may be due to the increasing number and depth of bifurcated crypts. Another study, by Rodrigues et al. [73], reported that rats fed with yacon flour plus *Bifidobacterium longum* (*B. longum*) had significantly higher tibia mineral content (calcium, magnesium, and phosphorus) and fracture strength.

As for human studies, the calcium concentration in blood was observed to be increased in the intervention group receiving yacon syrup as a secondary outcome in the study of Genta et al. [37]. However, there are inconsistencies in the effect of FOS on calcium absorption. Some studies have reported a positive effect of FOS on stimulating calcium absorption in adolescents, young men, and pre- and postmenopausal women [37,74], while one study showed no effect of consuming 15 g of FOS/day for 21 days in a group of healthy young men (*n* = 12) [75]. A possible reason for such discrepancies might be the small doses of FOS and inappropriate methods used to determine calcium absorption [75].

### 3.8. Adverse Effects

A study reported that subjects had side effects such as diarrhea, flatulence, nausea, and abdominal distension when consuming yacon at 0.29 g FOS/kg body weight/day [37]. These symptoms disappeared when the dose was reduced to 0.14 g FOS/kg body weight/day. In an animal study, no differences were found between low and high concentrations in the yacon-supplemented group with regard to adverse co5nsequences, except that cecal hypertrophy was observed in a few rats in the high-concentration yacon group [47]. There were two cases of severe adverse effects after consuming yacon. One was the case of a 55-year-old woman who suffered from anaphylaxis after ingesting yacon root [76], and the other was an animal study [77] that reported the development of renal lesions in rats with long-term consumption of yacon leaves.

Indicators of liver function in rats showed no significant difference after 28 days of a yacon diet, which indicates an absence of liver toxicity due to supplementation with yacon [73]. These findings were consistent with a study of 4 months of yacon flour supplementation (0.6% and 13% FOS) in rats [47].

## 4. Conclusions

Yacon has multiple nutrigenomic implications with regard to health outcomes. In addition, yacon is a useful resource for alternative and complementary prebiotics, for example, for intestinal health and for their antimicrobial and antioxidant effects. Although yacon has a long history as a root-based fruit in South America, future studies are needed to better elucidate its mechanisms and nutrigenomic properties regarding health outcomes. This is because there are limited data, especially on the safety evaluation of yacon. In addition, having a better understanding of the effects and mechanisms involved would allow yacon to be developed as a novel functional food as well.

## Figures and Tables

**Table 1:** Chemical composition of 1 kg fresh yacon root [13].

Variables	Mean
Dry matter (g)	115
Total carbohydrates (g)	106
Fructans (g)	62
Total free sugars (g)	26
*Free glucose (g)*	3.5
*Free sucrose (g)*	14
*Free fructose (g)*	8.5
Protein (g)	3.7
Fiber (g)	3.6
Fat (mg)	244
Calcium (mg)	87
Potassium (mg)	2282
Phosphorus (mg)	240

**Table 2:** An overview of major phytochemical compounds of yacon.

Parts of Yacon	Compounds/Nutrients Identified	Test Methods	References
Roots/tubers	Fructooligosacharides (1-kestose, nystose, and 1-fructofuranosyl nystose)	Fermentation by *Bifidobacterium and Lactobacillus*	Hermann et al. (1997) [13]; Niness et al. (1999) [14]; Roberfroid et al. (2010) [16]; Delgado et al. (2012) [4]; Paula et al. (2014) [15]
	Tryptophan	2,2′-azino-bis(3-ethylbenzothiazoline-6-sulphonic acid) (ABTS)	Sousa et al. (2015) [36]
	Chlorogenic acid	ABTS	Sousa et al. (2015) [36]
	Caffeic acid	ABTS	Sousa et al. (2015) [36]
	Ferulic acid	1,1-diphenyl-2-picrylhydrazyl (DPPH)	Simonovska et al. (2003) [5]
Leaves	Chlorogenic acid	Decoction DPPH and xanthine/xanthine oxidase (XOD) superoxide radical scavenging assays Ohmic-assisted decoction	Yan et al. (1999) [6]; Genta et al. (2009) [37]; Valentová et al. (2003) [9]; Simonovska et al. (2003) [5]; Khajehei et al. (2017) [38]
	Caffeic acid	Decoction DPPH and xanthine/XOD superoxide radical scavenging assays Ohmic-assisted decoction	Genta et al. (2009) [37]; Russo et al. (2015) [29]; Valentová et al. (2003) [10]; Khajehei et al. (2017) [38]
	Ferulic acid	DPPH and xanthine/XOD superoxide radical scavenging assays Ohmic-assisted decoction	Valentová et al. (2003) [10]; Khajehei et al. (2017) [38]
	Myricetin	Ohmic-assisted decoction	Khajehei et al. (2017) [26]
	Rutin	Decoction Ohmic-assisted decoction	De Andrade et al. (2014) [3]; Khajehei et al. (2017) [38]
	*ρ*-Coumaric acid	Ohmic-assisted decoction	Khajehei et al. (2017) [38]
	Gallic acid	Decoction	De Andrade et al. (2014) [3]
	Tryptophan	DPPH assay	Yan et al. (1999) [6]
	Enhydrin	Decoction	Genta et al. (2009) [37]
Flower	Myricetin	Decoction	De Andrade et al. (2014) [3]
	Gallic acid	Decoction	De Andrade et al. (2014) [3]

**Table 3:** An overview of major nutrigenomic properties of yacon.

Pharmacological Effects	Models Used	Parts/Forms of Yacon Used	References
Hypoglycemia effect	Streptozotocin-induced diabetic rats	Leaf extract	Aybar et al. (2001) [43]; Valentová and Ulrichová (2003) [9]
	Diabetic rats	Aqueous leaf extract	Simonovska et al. (2003) [5]; Barcellona et al. (2012) [39]
	Streptozotocin-induced diabetic and nondiabetic rats	Leaf extract	Baroni et al. (2008) [44]
	Streptozotocin-induced diabetic rats	Dried root extract	Satoh et al. (2013) [45]; Oliveira et al. (2016) [46]
	Normoglycemic, transiently hyperglycemic, and diabetic rats	Leaf extract	Genta et al. (2009) [37]
	Decoction and enhydrin-fed Wistar rats	Leaf extract	Barcellona et al. (2012) [39]
	Humans	Freeze-dried powder	Scheid et al. (2014) [11]
	Humans	Syrup	Genta et al. (2009) [37]
Hypolipidemic effect	Normal and streptozotocin-induced diabetic rats	Dried root flour	Genta et al. (2005) [47]; Habib et al. (2011) [48]
	Hypercholesterolemic male Wistar rats	Root extract	Oliveira et al. (2016) [46]; Oliveira et al. (2013) [49]
	Mildly dyslipidemic premenopausal women		Genta et al. (2009) [37]
Anti-inflammatory effects	Hypercholesterolemic rats	Root extract	Oliveira et al. (2016) [46]
	Adult male BALB/c mice	Leaf extract	Inoue et al. (1995) [34]; Lin et al. (2003) [7]; Schorr et al. (2007) [50]; Oliviera et al. (2013) [49]
Antimicrobial effects	*P. oryzae*	Leaf extract	Inoue et al. (1994) [34]
	*B. subtilis*	Leaf extract	Lin et al. (2003) [7]
	Gram-positive organisms (*Staphylococcus aureus, Staphyilococcus epidermidis, and Bacillus subtilis*)	Leaf extract	Padla et al. (2012) [51]
